# Effect of Surface Morphology and Texture of Short-Tailed Shrew’s Toe on Tribological Properties of 65Mn Steel

**DOI:** 10.3390/biomimetics10090631

**Published:** 2025-09-18

**Authors:** Yachao Zhang, Jian Zhang, Wengang Chen, Haijun Wang, Zhaoling Qiu, Wen Wang, Yali Zhang, Dongyang Li

**Affiliations:** 1 School of Machinery and Transportation, Southwest Forestry University, Kunming 650224, China; zhangyachao@163.com (Y.Z.);; 2 Academician Dongyang Li Workstation in Yunnan Province, Kunming 650224, China; 3 School of Intelligent Manufacturing, Panzhihua University, Panzhihua 617000, China; 4 School of Materials and Chemical Engineering, Southwest Forestry University, Kunming 650224, China; 5 Department of Chemical and Materials Engineering, University of Alberta, Edmonton, AB T6G2H5, Canada

**Keywords:** bionic texture, laser processing, 65Mn steel, friction wear, friction coefficient

## Abstract

To reduce the friction coefficient and wear in tillage machinery during operation, biomimetic textures with different densities inspired by the short-tailed shrew’s claw were designed using biomimetic principles. These textures were applied to the surface of 65Mn steel using laser processing technology. This study investigated the effects of these bionic textures on the tribological properties of 65Mn steel surfaces in two environments: dry friction and soil friction. Friction and wear tests were conducted, and the friction coefficient, wear morphology, and wear quality were measured using a friction and wear testing machine, a scanning electron microscope (SEM), and a three-dimensional profilometer. The results indicate that under dry friction conditions, the tribological properties of specimens with bionic textures were significantly improved compared to non-textured specimens. The frictional properties of the specimens with bionic textures were optimized at a texture density of 20%, with an average coefficient of friction reduction of 24%. Under soil friction conditions, the samples with bionic textures demonstrated better tribological performance at densities of 20% and 30% compared to the non-textured samples, with decreases in the average coefficient of friction of 1.3% and 2.9%. The special surface structure of the bionic short-tailed shrew claw can effectively reduce friction heat effects and wear, demonstrating significant anti-friction and anti-wear performance.

## 1. Introduction

In the development process of modern agriculture, enhancing the mechanization level plays a crucial role, and enhancing the performance of agricultural machinery becomes the key to achieving this goal [1]. For a long time, the high operating resistance and significant wear-and-tear problems of agricultural machinery have restricted its performance. Therefore, researchers’ main research objectives and directions include reducing the operating resistance of agricultural machinery and enhancing its wear resistance. For this reason, 65Mn steel, with its excellent hardness, good toughness, excellent wear resistance, excellent hardenability, and other characteristics, has become the material of choice for manufacturing agricultural machinery, automobiles, ships, railroad transportation, industrial machinery, and other key components [2].

In the field of agriculture, the application of bionics mainly focuses on research areas such as de-attachment, drag reduction, and viscosity reduction related to agricultural machinery, where the rough claw toes of soil animals are the most commonly used bionic prototypes in research on bionic drag reduction and reducing the wear of cultivation components [3,4]. Studies reveal that in nature soil-digging animals such as moles [5], pangolins [6], badgers [7], and earthworms [8] demonstrate excellent digging abilities and effectively reduce soil adhesion by virtue of their unique claw toe morphologies and biomechanical properties, reflecting their unique wisdom of survival. These properties have been intensively studied and applied to soil-touching components of agricultural machinery, such as plows, deep-pine shovels, furrow openers, rotary tillage knives, and so on. In addition, when dung beetles are in the process of digging and when tumbling dung balls, desert lizards, and pangolin beetles perform long-term movements in sandy soil, their body structures show good abrasion resistance and drag reduction, and these properties are often borrowed to improve the abrasion resistance and drag reduction of agricultural soil-touching implements in the process of plowing [9]. As a result, abrasion resistance and drag reduction, two aspects that have become the earliest and most successful areas of bionics research, are applied to agricultural machinery.

In recent years, surface-texturing technology, as a means of surface modification, has been widely used in a variety of fields such as medicine, computers, automobiles, etc. [10], and has also shown its importance in designing the functional structure and appearance of agricultural machinery. Numerous researchers have pointed out that by constructing appropriate textures on the surfaces of materials, the friction coefficient can be effectively reduced and the wear resistance of materials can be enhanced, thus increasing their working efficiency and prolonging their service lives [11]. Inspired by the structure of pangolin scales, Wu Keyan et al. [12] used laser processing technology to prepare earthworm-like head pits and dung beetle-like head grooves on the surface of 65Mn steel. When the texture rate was 20%, the friction coefficient decreased by 21.8% and 47.3%, respectively, and the wear rate decreased by 18.0% and 28.8%, respectively. Ma Yunhai et al. [13] conducted friction and wear experiments on transverse and longitudinal texture patterns with soil abrasives using pangolin scales. The results showed that the texture direction and sliding direction of the pangolin scales had a significant effect on the friction and wear performance, and when the sliding speed of the abrasive was reduced, the ratio of the amount of wear increased accordingly. Further comparisons revealed that the wear amount of pangolin scale specimens with transverse textures was about two to three times that of specimens with longitudinal textures, which indicated that the longitudinal textured morphology had superior performance in terms of abrasion resistance compared to the transverse textured morphology. Liu et al. [14] scanned the surface of the corrugated body of an earthworm using a 3D laser scanner, extracted the surface texture curves, 3D-printed biomimetic specimens using a photosensitive resin as a raw material, and used the specimens to investigate the drag-reduction performance of soil joint components used for lubrication and drag reduction. The experimental results show that the bionic samples have excellent drag-reduction performance, and the drag-reduction rate reaches 22.65% to 34.89%.

Li [15] prepared pits with different densities on the surface of 45 steel. In this study, the effects of the pit density on friction and wear performance during dry friction and insufficient oil lubrication were tested using a friction and wear testing machine. The results showed that during insufficient oil lubrication and dry friction, the wear rate of the textured samples was lower. As the texture density increased, the wear rate first decreased and then increased, and the wear resistance was best at a density of 8.1%.

Zhong [16] used laser processing technology to prepare specimens with hexagonal textures on the surface of AISI-1045 steel to study the mechanism by which geometric texture characteristics and operating parameters influence friction performance. The results showed that compared with smooth surfaces, surfaces with hexagonal textures exhibited superior friction-reduction performance. Among them, the friction coefficient of specimens with a surface texture density of 25% was reduced by 41%.

Li [17] processed groove micro-textures on the surface of 316L stainless steel. Friction and wear test results showed that, compared with non-textured samples, the friction coefficient of textured samples decreased and wear resistance improved. When the groove width was 100 μm and the spacing was 200 μm, the friction-reduction and wear-resistance performance was optimal.

Based on the theory of biomimetic tribology, in this study biomimetic textures with friction-reducing and wear-resistant properties were designed and prepared by imitating the microstructures of biological surfaces, a technique that has been widely used in the field of agricultural engineering. In order to enhance the friction-reducing and wear-resistant properties of agricultural touchdown machinery and extend its service life, this study simulated the surface texture characteristic curve of the clawed toe of the short-tailed shrew, and based on this, bionic microstructures with different distribution densities were designed. Bionic micro-textures were prepared on the surface of 65Mn steel by laser processing technology. By carrying out friction and wear experiments, this study investigated the effect of bionic micro-textures on the tribological properties of 65Mn steel surfaces under both dry friction and soil friction conditions. The results of this study are intended to provide theoretical support for the application of bionic micro-textiles in industrial and agricultural fields, with the aim of reducing frictional resistance, minimizing wear loss, enhancing work efficiency, extending equipment life, and conserving natural resources.

## 2. Materials and Methods

### 2.1. Test Materials

In this study, 65Mn steel was selected as the experimental base material, and its chemical composition is detailed in Table 1. Laser cutting equipment was utilized to cut a 65Mn steel plate into test samples with dimensions of 50 × 50 × 10 mm.

### 2.2. Study to Select Parameters and Preparation Methods for Bionic Texture Structure

**Figure 1:**
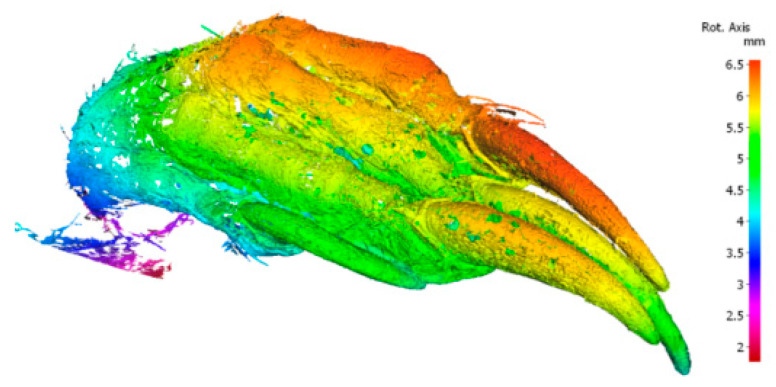
A three-dimensional scan of the right anterior claw toe of a mole shrew (short-tailed shrew).

**Figure 2:**
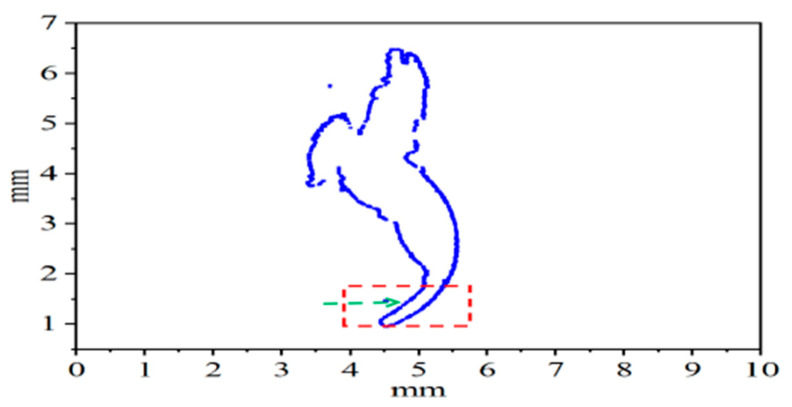
Claw and toe contour curve morphology.

The short-tailed shrew, as a typical class of soil-burrowing animal, exhibits excellent soil-cutting and digging abilities. The special structure of its claw toes endows it with a significant physical strength-saving effect during the digging process, thus realizing the goal of saving energy and reducing drag. In this study, the three middle right front claw toes of the short-tailed shrew were selected as samples for morphological feature extraction. Coordinate data of the central inner and outer longitudinal ridges of each toe were collected, and morphological maps of the claw toes were generated using Origin software. By analyzing the claw toe morphology data, the fingertip portion was further filtered for feature curve fitting. Figure 1 shows the 3D-scanned morphology of the right claw toe of the short-tailed shrew, while Figure 2 shows the morphology of the claw toe generated by Origin software. The red area in the figure is labeled with the filtered morphology data of the fingertip part.

**Figure 3:**
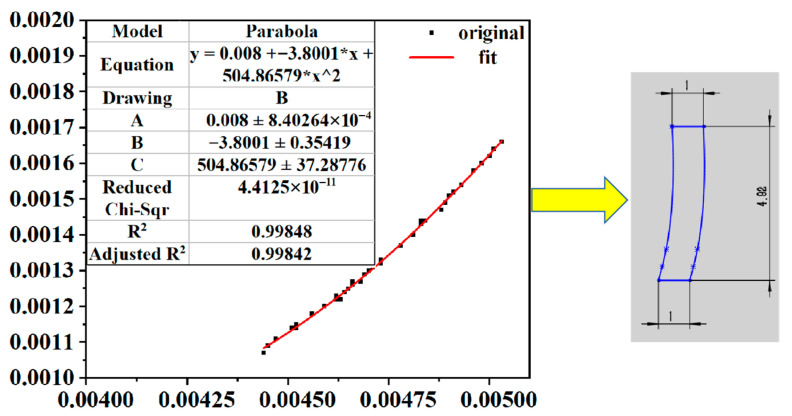
The characteristic fit of the curve within toe 3.

In this study, coordinate data were collected for the central medial and lateral longitudinal ridges of each toe, which in turn generated paw–toe morphometric maps. Based on these claw toe morphometric data, specific samples were selected for curve-fitting analysis. Six fitted characteristic curves were constructed for the inner and outer sides of the three middle toes, resulting in fitting equations (see Table 2) and their decision coefficients for six curves. The resulting coefficients of determination ranged from 0.95765 to 0.99842, and all were located within the (0,1) interval and were close to 1, indicating that the fitted curves were in high agreement with the expectations and ensured a fit with high accuracy. Figure 3 demonstrates the fitted characteristic plot of the medial toe 3 curve with the fitting polynomial Y3n = 0.008 + (−3.8001*x) + 504.86579*x^2^ and a coefficient of determination (R^2^) of 0.99842. Therefore, the medial toe 3 curve was selected as the texture curve.

### 2.3. Preparation of Bionic Texture

A heat treatment involving quenching and tempering was carried out on the small square of a 65Mn specimen, and after cleaning, industrial ethanol was used to remove the oil on the surface in an ultrasonic cleaner. Surface defects and the oxidized layer were removed with a metallographic grinding and polishing machine. Then, a polishing treatment was carried out, where 400 #, 600 #, 800 #, 1000 #, 1200 #, 1500 #, 2000 #, and 2500 # SiC sandpaper were used for polishing, followed by a metallographic polishing flannel and a W0.5 diamond polishing paste. The small square of the sanded specimen was polished to a mirror state, 75% of the industrial ethanol in the ultrasonic cleaner was used for cleaning, and finally the specimen was blown dry with a hair dryer and sealed with oil for use.

**Figure 4:**
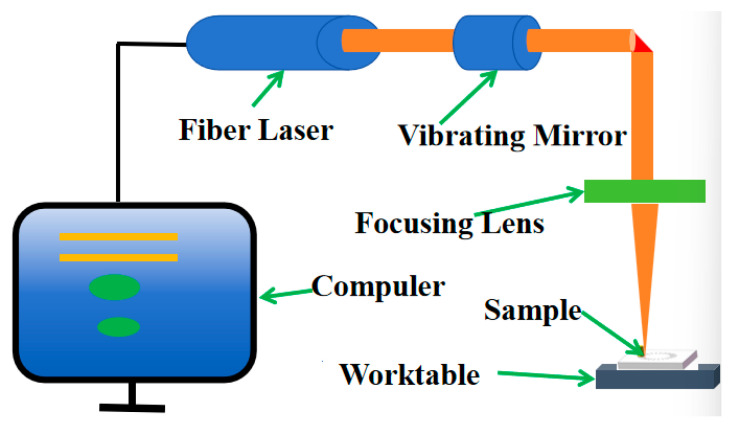
Diagram of texture preparation system.

**Figure 5:**
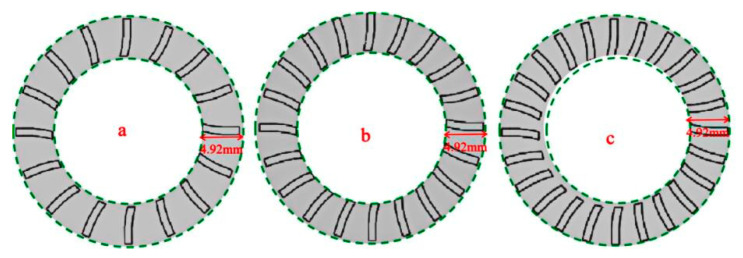
Organizational distribution ((**a**–**c**) 20%, 25%, and 30%).

The three inner curves were enlarged to a length of 4.92 mm and shifted by 1 mm, and their lower positions were connected to form a closed pattern, which served as the texture pattern. While preparing the surface texture of the 65Mn steel, a Grace X 355-3 Alaser (documented in Table 3) from Suzhou Qishan Automation Equipment Co. Ltd., China, was used. It possessed an optical power of 3 W. Parameters integral to this procedure included the frequency of 30 kHz, the laser wavelength of 355 nm, and the pulse temporal span of 25 Ms. Figure 4 presents a schematic of the procedure, which was orchestrated by a computer apparatus, where leverage was found through fiber laser incidents generating coaxial beam emissions. These emissions were reflected onto subsequent surfaces via oscillating mirrors, and conducted reflection was performed using a condenser mirror [18]. Then, this beam intersected the sample plane, resulting in the three biomimetic textures illustrated in Figure 5. All textures had unique orientations and possessed depths that were quantitatively assessed at 50 m, while the textural occupation was set at ratios like 20%, 25%, and 30%. Observably, burs formed due to heightened energetic pulses from the laser. They were addressed through the requisite deployment of a PGlS metallographic polisher. Post-protrusion removal involved thorough cleansing, where samples were submerged in absolute ethanol for ten minutes and rigorous purification was performed using a CJ-009S ultrasonic instrument.

### 2.4. Friction Wear Test

**Figure 6:**
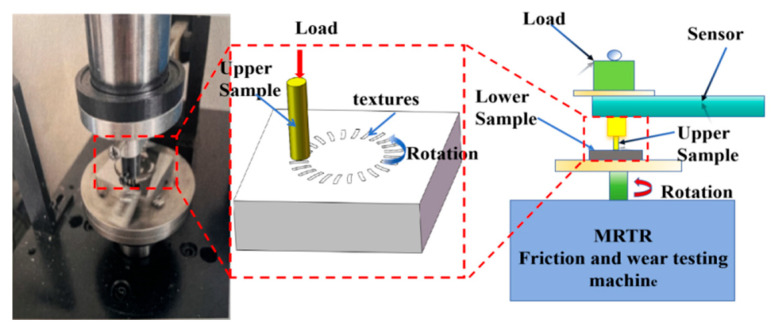
Schematic diagram of friction wear tester.

An MRTR multifunctional friction and wear tester was utilized to conduct experiments (Figure 6). The friction method employed was rotary friction, with the upper specimen fixed and the lower specimen undergoing repeated rotation. The upper specimen consisted of cylindrical GCr15 bearing steel measuring 5 mm in diameter and 15 mm in height; its specific composition is detailed in Table 4. The friction pair configuration was of the pin–disk type.

The ambient temperature was 25 °C, the air humidity was 71%, the soil moisture content was 18.5%, and the soil particle size data are shown in Table 5.

To minimize the likelihood of inconsistent test results, in this study three replicate tests were conducted for each control group, ensuring the consistency of the testing conditions. Before and after each test, the specimens were cleaned and weighed, with the relevant data being recorded. The test parameters (see Table 6) were established with a rotation radius of 10 mm, a rotational speed of 200 rpm, an applied load of 10 Newtons, and a test duration of 120 min. The wear morphology of the specimens was observed using an optical microscope to investigate the impact of varying surface texture densities on the friction performance of the specimens under both dry and soil friction conditions.

## 3. Test Results and Analysis

### 3.1. Surface Friction Properties of 65Mn Steel Under Dry Friction Conditions

**Figure 7:**
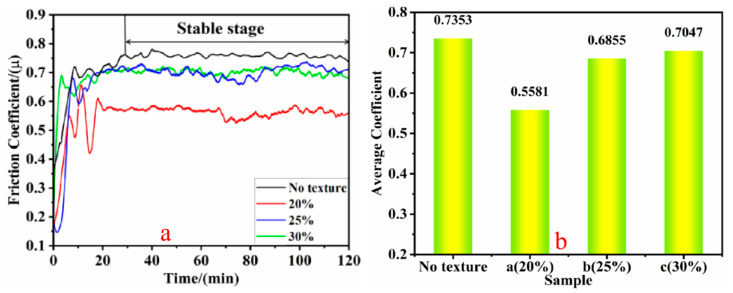
Friction coefficients of specimens with different texture densities during dry friction ((**a**) friction coefficient curve and (**b**) average friction coefficient).

The variation curves of the friction coefficients of specimens with different texture densities under dry friction conditions are shown in Figure 7. At the beginning of the test (0–25 min), the friction coefficient of each specimen increased rapidly with significant fluctuations. As the test progressed, the wear stabilization stage (30 min to 120 min) was reached, the curves tended to stabilize, and the fluctuation of the friction coefficient decreased. The friction coefficients of the textured specimens were significantly lower than those of the non-textured specimens. The inability to expeditiously remove abrasive particulates during wear resulted in considerable accumulation at the frictional boundary. An intensification of abrasive wear that elevated the mean coefficient of friction and induced significant variability within the frictional response curve was evident. Figure 7a indicates that the friction coefficient of the texture-free specimen was the highest and exhibited greater fluctuation. The lowest friction coefficient was observed for the textured specimen with a density of 20%. It continued to decrease after 70 min with notable fluctuations. This is because, under dry friction conditions, as friction progresses, an oxide layer gradually forms on the surface. The incompleteness of the oxide layer allows abrasive particles to more easily enter pores, thereby reducing the contact area. At the same time, the microstructure of a textured surface can capture and store these abrasive particles, further reducing friction. Therefore, these two mechanisms work synergistically under dry friction conditions, jointly leading to a decrease in the coefficient of friction [18]. The friction coefficients of the textured specimens with densities of 25% and 30% tended to stabilize at the beginning of the wear stabilization stage, but as the test time increased, the friction coefficients of the 25% specimens were higher than those of the 30% specimens. Both were significantly lower than those of the non-textured specimens. This could be due to the intense wear of the surface texture and the filling effect of abrasive debris, which weakens the texture’s ability to hold debris, increases the contact area during the friction process, and gradually increases the roughness of the contact area, resulting in a certain upward trend in the friction coefficient [19]. In Figure 7b, the average coefficients of friction are 0.7353 for the non-textured specimen (µ_non_), 0.5581 for the textured specimen at 20% density (µ_20%_), 0.6855 for the textured specimen at 25% density (µ_25%_), and 0.7047 for the textured specimen at 30% density (µ_30%_). Comparative results show that µ_20%_ < µ_25%_ < µ_30%_ < µ_non_. This outcome reveals that the friction coefficients of the different densities of the specimens with bionic textures were reduced by 24% (20% density), 6.8% (25% density), and 3.1% (30% density) compared to the non-textured specimens. It is evident that the three samples with biomimetic textures with different distribution densities significantly reduced friction and could reduce the range of friction coefficient fluctuations. This suggests that employing bionic texture technology to reduce the friction and wear of mechanical surfaces is feasible and that the distribution density of the texture is one of the key factors influencing the friction-reduction effect.

### 3.2. Surface Friction Properties of 65Mn Steel Under Soil Friction Conditions

**Figure 8:**
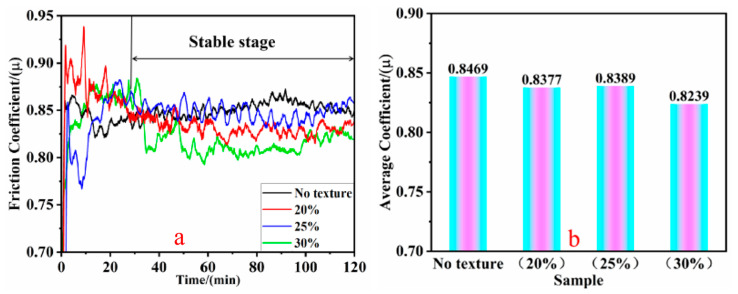
Friction coefficients of specimens with different texture densities during soil friction ((**a**) friction coefficient curve and (**b**) average friction coefficient).

Figure 8 depicts the fluctuations of the friction coefficients of the specimens with bionic textures with varying densities under soil friction conditions. In Figure 8a, it is evident that the friction coefficients of the specimens with bionic textures with densities of 20% and 30% were lower than those of the non-textured specimens in the wear stabilization stage. Conversely, the friction coefficients of the specimens with bionic textures with densities of 25% started higher than those of the non-textured specimens in the wear stage but decreased as the experiment progressed. The friction coefficient curves of the three specimens with bionic textures exhibited more pronounced fluctuations during the wear stage (0–25 min); however, as the experiment continued, the curves tended to decline in the wear stabilization stage (30–120 min), indicating a certain friction-reducing effect. Figure 8b presents their average friction coefficients as follows: µ_Non_ = 0.8469, µ_20%_ = 0.8377, µ_25%_ = 0.8389, and µ_30%_ = 0.8239, where µ_30%_ < µ_20%_ < µ_25%_ < µ_Non_. These outcomes suggest that the specimens with bionic textures outperformed the non-textured specimens in terms of friction under soil friction conditions, with friction coefficients that were reduced by 1.3%, 0.9%, and 2.9%, respectively. On the one hand, this is because in soil environments, friction pairs continuously come into non-uniform contact with soil particles of different shapes and sizes, causing the entire friction process to be in an unstable state. Secondly, biomimetic textures have the function of storing abrasive soil particles. During design, to better facilitate the movement of the short-tailed shrew claws in soil, each texture was connected end-to-end, thereby serving to both store abrasive particles and allow particles to circulate within the texture, reducing particle accumulation and abrasive wear, and ultimately achieving the goal of lowering the friction coefficient. On the other hand, the unique shape and arrangement of the biomimetic texture created specific convex and concave patterns on the substrate surface. When in contact with the opposing component in a soil environment, this effectively reduced the actual contact area [20].

**Figure 9:**
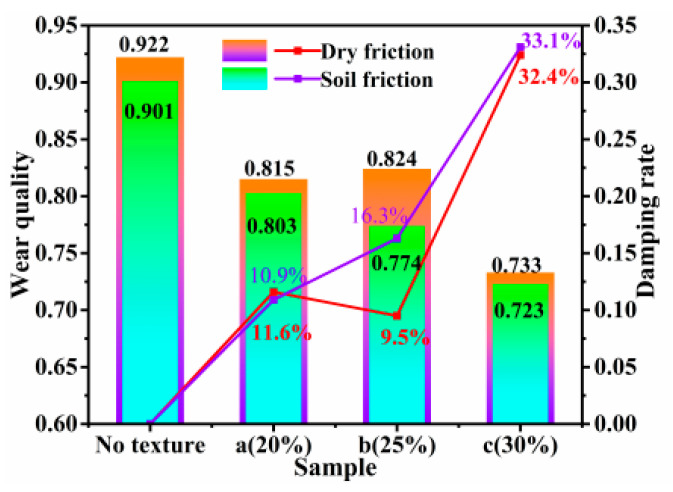
Wear quality of each sample.

Figure 9 reveals a comparison of the wear amount of each specimen under two different working conditions. In the graphs, it can be seen that the wear amount of the non-textured specimens reached the maximum value in both working conditions. Comparatively speaking, the abrasion amount of the specimens with bionic textures was reduced, indicating that the bionic texture possesses certain abrasion resistance. Specifically, in the dry friction condition, the abrasion amounts of the textured specimens decreased by 11.6%, 9.5%, and 32.4%, respectively, while in the soil friction condition, the abrasion amounts of the textured specimens decreased by 10.9%, 16.3%, and 33.1%, respectively. This phenomenon is mainly attributed to the “microstorage” effect of the bionic texture, which can effectively capture abrasive fragments and collect soil particles in a timely manner, thus reducing the contact area between the upper and lower specimens and further decreasing the abrasion of the specimen surface [21]. The bionic texture also effectively disperses the stress concentration during friction and prevents excessive localized wear. This “microstorage” effect can be further enhanced by optimizing the texture parameters, such as the shape, depth, and distribution density, to further improve the wear resistance of 65Mn steel under soil friction conditions.

When comparing the friction coefficient curves under both conditions, it is evident that the friction coefficient of each specimen was markedly higher in the soil friction condition than during dry friction and that the fluctuation amplitude was more pronounced. This was because in the soil friction condition, a multitude of soil particles adhered to the surface of the woven specimen, resulting in incomplete contact between the grinding pin and the specimen surface. Consequently, the contact area was relatively small, which increased the stress on the contact surface and intensified the degree of wear, leading to a rapid rise in the coefficient of friction [22]. A further comparison of the wear quality under both conditions reveals that the wear under the dry friction condition was greater than that under the soil friction condition. In both conditions, the specimen with a texture density of 30% exhibited the best anti-wear effect. This was because the higher texture density exacerbated wear on the friction surface, but simultaneously, the denser texture distribution allowed the texture to store more abrasive debris, thereby reducing the abrasive wear on the surface of the specimen, which significantly diminished the wear of the textured specimen.

### 3.3. Sample Wear Morphology Analysis

**Figure 10:**
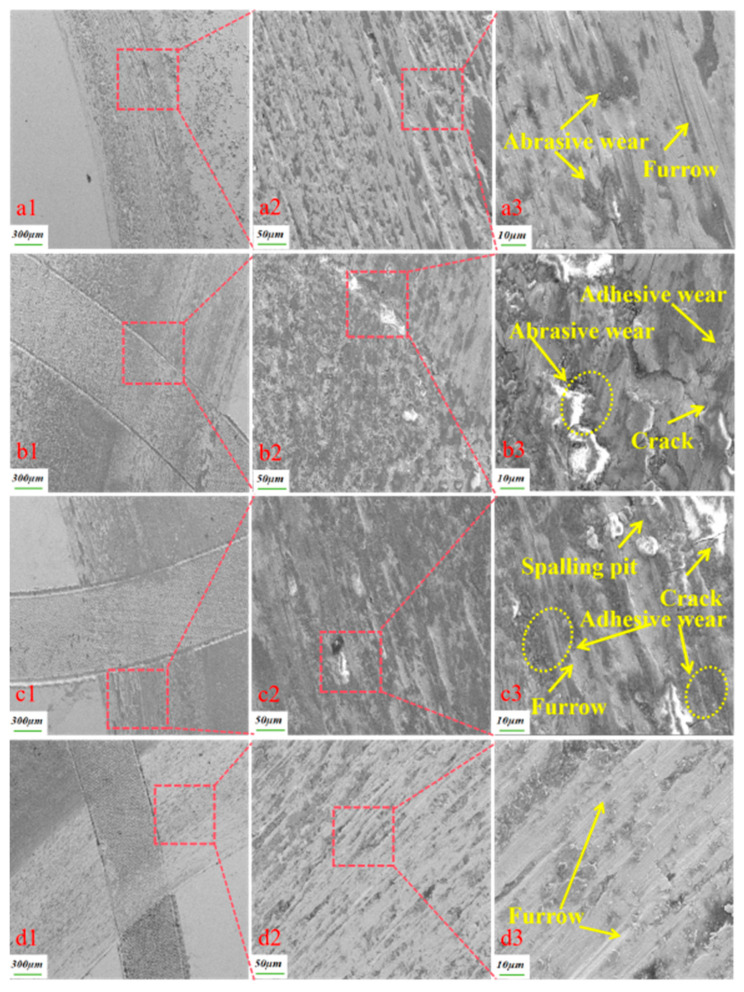
SEM wear patterns under dry friction conditions. ((**a1**) the local wear morphology of the non-textured specimen at a magnification of 300 µm, (**a2**) the wear morphology at a magnification of 50 µm within the red box in figure (**a1**), (**a3**) the wear morphology at a magnification of 10 µm within the red box in figure (**a2**), (**b1**) the local wear morphology of a sample with a texture density of 20% at a magnification of 300 µm, (**b2**) the wear morphology within the red box in (**b1**) at a magnification of 50 µm, (**b3**) the wear morphology within the red box in (**b2**) at a magnification of 10 µm, (**c1**) the local wear morphology of a sample with a texture density of 25% at a magnification of 300 µm, (**c2**) the wear morphology of the red box in (**c1**) at a magnification of 50 µm, (**c3**) the wear morphology of the red box in (**c2**) at a magnification of 10 µm, (**d1**) the local wear morphology of a sample with a texture density of 30% at a magnification of 300 µm, (**d2**) the wear morphology within the red box in figure (**d1**) at a magnification of 50 µm, (**d3**) the wear morphology within the red box in figure (**d2**) at a magnification of 10 µm).

Figure 10 displays microscopic images of the wear patterns of various specimens under dry friction conditions. When observing the wear morphology of the specimen without texture treatment in Figure 10a3, it is evident that the surface is covered with wear marks and furrow-like abrasions, which are primarily characterized by adhesive and granular wear. This type of wear is attributed to the adhesion of the upper and lower specimen surfaces during sliding. As the test progressed, the adhesion points were subjected to repetitive shear forces, resulting in the formation of abrasive particles [23]. As the upper and lower specimens slid against each other, the lower specimen experienced pressure, causing abrasive grains to embed in its surface. The friction effect led to the creation of furrow-like abrasion marks on the surface as the test progressed [18]. In contrast, the textured specimens with weaving densities of 20%, 25%, and 30%, as shown in Figure 10b3–d3, exhibit fewer furrows on their surfaces, smaller areas of particle abrasion, and significant accumulations of abrasive material in the furrows. Only a small amount of abrasive debris adhered to the surface of the friction vice, with a large quantity accumulating in the inner rim of the friction vice. The abrasive chips generated between the friction vice drove most of the abrasive chips into the textured pits during the rotary motion of the pair of abrasive pins, which led to reduced abrasive particle wear on the textured specimens and thus significantly diminished the degree of wear on the textured specimens. The plough furrows on the surface of the fabricated bionic specimens are lighter than those on the non-textured specimens because the bionic textures can store abrasive debris and can reduce abrasive grain wear and adhesive wear. The surface of the textured sample has similar wear marks, but due to the presence of the texture on the surface, the wear marks are less severe [24]. Adhesive wear is the primary wear mechanism under dry friction conditions [25].

**Figure 11:**
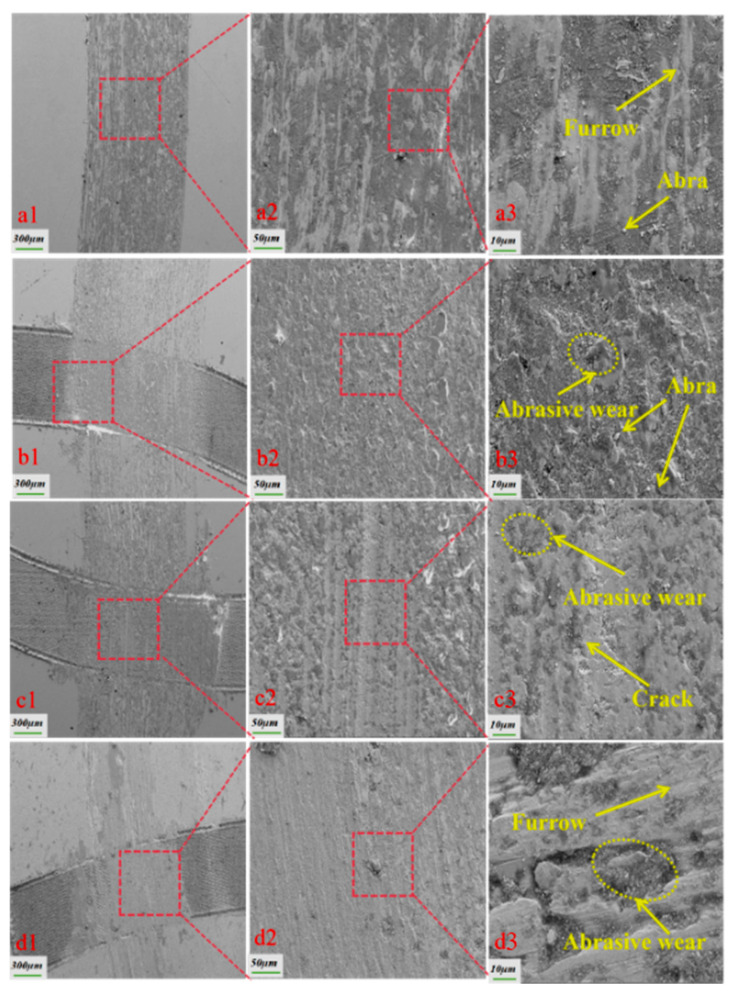
SEM wear patterns under soil friction conditions. ((**a1**) the local wear morphology of the non-textured specimen at a magnification of 300 µm, (**a2**) the wear morphology at a magnification of 50 µm within the red box in figure (**a1**), (**a3**) the wear morphology at a magnification of 10 µm within the red box in figure (**a2**), (**b1**) the local wear morphology of a sample with a texture density of 20% at a magnification of 300 µm, (**b2**) shows the wear morphology within the red box in (**b1**) at a magnification of 50 µm, (**b3**) the wear morphology within the red box in (**b2**) at a magnification of 10 µm, (**c1**) the local wear morphology of a sample with a texture density of 25% at a magnification of 300 µm, (**c2**) the wear morphology of the red box in (**c1**) at a magnification of 50 µm, (**c3**) the wear morphology of the red box in (**c2**) at a magnification of 10 µm, (**d1**) the local wear morphology of a sample with a texture density of 30% at a magnification of 300 µm, (**d2**) shows the wear morphology within the red box in figure (**d1**) at a magnification of 50 µm, (**d3**) shows the wear morphology within the red box in figure (**d2**) at a magnification of 10 µm).

Figure 11 illustrates the wear morphologies of various specimens under soil friction conditions. In Figure 11a3, it is evident that the texture-free specimen exhibits more severe wear, characterized by a multitude of particle abrasions and scratches on the surface, along with extensive adhesive wear. In contrast, Figure 11b3,c3 reveal that the textured specimen has less severe wear, with fewer scratches and adhesive wear areas. However, significant amounts of abrasive debris and soil particles accumulated in the texture pits, which aided in minimizing the overall wear of the specimen. Further analysis of Figure 11b3,c3 indicates that cracks and spalling are present on the surface of the textured specimen. This is attributed to the continuous rubbing of fine soil particles against the lower specimen around the friction vice during the soil friction process, leading to spalling of some substrate material and cracks as the test progressed. Figure 11d3 demonstrates that when the texture density was high, the bionic texture increased the surface wear of the friction sub-surface; conversely, the texture-free specimen tended to be extruded to the edges of the soil particles under load due to its larger contact area with the pins. The pits on the surface of the textured specimen could store soil particles, and as the wear time progressed, the texture gradually wore down, allowing the soil particles within the pits to re-enter between the friction sub-surfaces. The abrasive debris produced by the texture-free specimen during the wear process could not be easily discharged, resulting in abrasive wear between the friction partners, which exacerbated the loss of the specimen surface. It can be clearly seen in Figure 11a3 that the abrasive debris generated between the friction vice drove the abrasive debris into the texture pits during the rotation of the pin, which reduced the wear on the surface of the friction vice and thus reduced the wear of the textured specimen [26]. This suggests that an appropriate texture density can reduce the friction factor of the friction sub-surface and decrease wear, effectively enhancing the friction and wear performance of the friction sub-surface. However, an excessively high texture density may result in increased wear on the friction sub-surface because a higher texture density reduces the spacing between the fabrics, causing soil particles to be packed more densely among the friction sub-surfaces, which increases contact stresses and the wear rate between the friction sub-surfaces [27].

### 3.4. Three-Dimensional Morphology Analysis of Specimens

**Figure 12:**
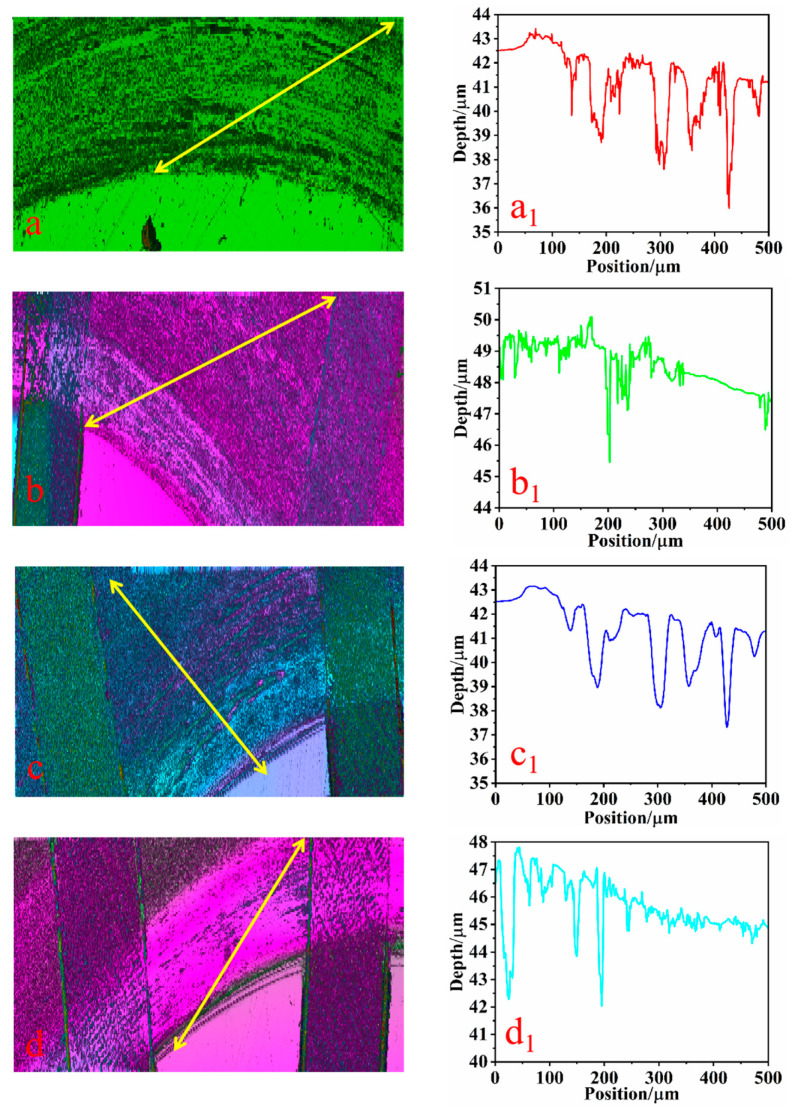
Three-dimensional morphology of wear depth in dry friction condition. ((**a**) the local three-dimensional morphology diagram of the sample without texture, (**a1**) the wear depth trajectory diagram of the area indicated by the arrow in figure (**a**), (**b**) the local three-dimensional morphology diagram of the sample with 20% texture, (**b1**) the wear depth trajectory diagram of the area indicated by the arrow in figure (**b**), (**c**) the local three-dimensional morphology diagram of the 25% textured specimen, (**c1**) the wear depth trajectory diagram of the area indicated by the arrow in figure (**c**), (**d**) the local three-dimensional morphology diagram of the 30% textured specimen, (**d1**) the wear depth trajectory diagram of the area indicated by the arrow in figure (**d**)). The arrows indicate the wear scratch data collected from a 500 μm area of the cross-section of each sample’s scratch, which is used to assess the wear depth.

**Figure 13:**
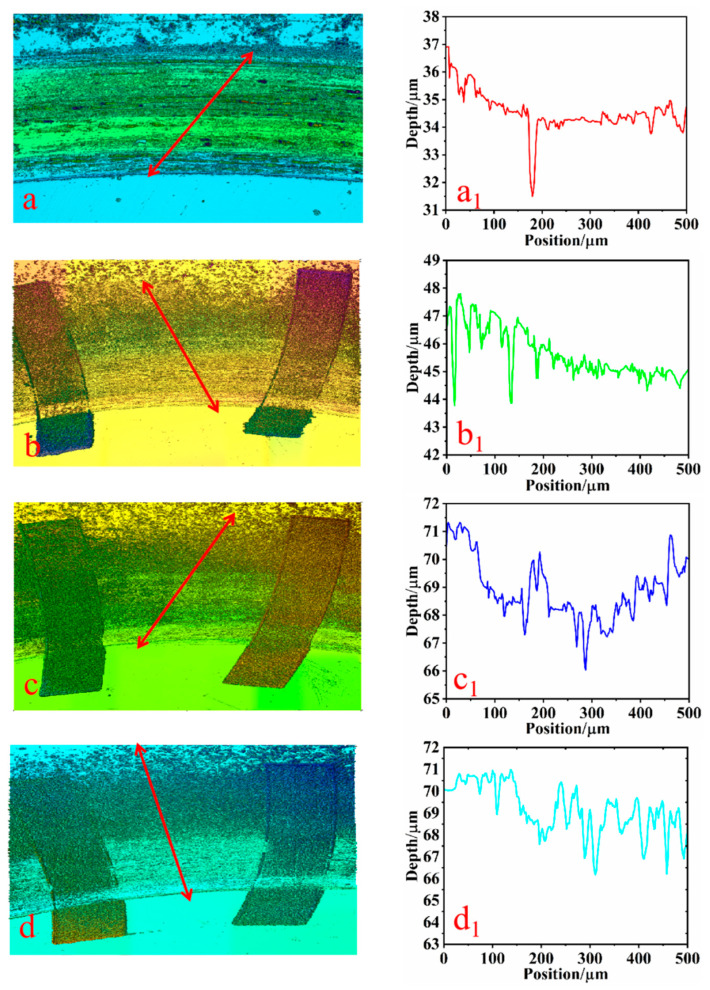
Three-dimensional morphology of wear depth in soil friction condition. ((**a**) the local three-dimensional morphology diagram of the sample without texture, (**a1**) the wear depth trajectory diagram of the area indicated by the arrow in figure (**a**), (**b**) the local three-dimensional morphology diagram of the sample with 20% texture, (**b1**) the wear depth trajectory diagram of the area indicated by the arrow in figure (**b**), (**c**) the local three-dimensional morphology diagram of the 25% textured specimen, (**c1**) the wear depth trajectory diagram of the area indicated by the arrow in figure (**c**), (**d**) the local three-dimensional morphology diagram of the 30% textured specimen, (**d1**) the wear depth trajectory diagram of the area indicated by the arrow in figure (**d**)). The arrows indicate the wear scratch data collected from a 500 μm area of the cross-section of each sample’s scratch, which is used to assess the wear depth.

The three-dimensional morphology of the wear on the surface of each specimen is depicted in Figure 12 and Figure 13, which illustrate the specimens’ three-dimensional morphologies and surface wear depths under dry friction and soil friction conditions, respectively. Figure 12a–d and Figure 13a–d represent the microscopic morphologies of the specimens with texture densities of 0%, 20%, 25%, and 30%, respectively. And they depict the corresponding wear depths. In this study, Gwyddion software was utilized to gather wear data from the scratched areas, with the regions indicated by the arrows (a, b, c, and d) chosen as references for wear data collection. Wear scratch data with a length of 500 μm were extracted under both working conditions to assess the wear depths. Subsequently, line graphs of the wear depths (a1, b1, c1, and d1) were generated using the plotting tool in Origin software.

Upon examining Figure 12a, it is evident that the surface of the non-textured structure exhibits numerous defects, including scratches, grooves, and pits. These imperfections are densely packed with a clustered distribution. Furthermore, Figure 12a1 reveals that the depth of these abrasions is approximately 7 μm, indicating a severe level of wear. However, it is apparent that when varying densities of texture patterns were applied to the surfaces of the specimens, as depicted in Figure 12b–d, the surface defects such as scratches, grooves, and pits were somewhat diminished. In Figure 12b1–d1, it is noted that the wear depths of the woven specimens all decreased. Specifically, the wear depth of the specimen with a texture density of 20% is around 4 µm. For the texture density of 25%, it is approximately 5 µm; and for the texture density of 30%, it is also roughly 5 µm. The wear depth of the texture-treated specimens is notably less than that of the non-textured specimens, which have a wear depth of 7 µm, suggesting that the introduction of the texture indeed conferred a wear-resistant benefit. This observation aligns with the reduction in wear volume for each specimen shown in Figure 8. Additionally, the texture structure is clearly filling with abrasive chips, which further prevents secondary damage from hard abrasive particles. Consequently, a higher texture density can accommodate more abrasive debris, contributing to a reduction in wear [28].

As depicted in Figure 13a, under soil friction conditions, the surface of the non-textured specimen exhibited a multitude of dense scratches, accompanied by flaking of the metal layer. The entire scratch wear area appeared incomplete. Figure 13a1 indicates that the maximum scratch depth of the non-textured specimen was approximately 6 μm. Upon processing the specimen’s surface with bionic fabrics of varying densities, it was observed that the scratches on the specimen’s surface gradually diminished, the peeling of the metal layer was mitigated, and the wear area of the scratches remained relatively intact. Additionally, the depth of the scratches showed a tendency to decrease gradually. Figure 13b1–d1 further illustrate that the depth of the scratches decreased significantly, with the wear depths being about 3 μm at a texture density of 20%, 4 μm at 25%, and 3 μm at 30%. In summary, despite the texture’s strong ability to trap abrasive chips, the reduction in the actual contact area led to increased contact stress, causing stress concentration in the smooth areas. This further aggravated wear and caused the texture structure to flatten and lose its ability to capture abrasive chips [29]. Consequently, only when the texture density is optimal can the contact stress and the debris-capturing ability be adequately balanced, achieving the texture’s anti-wear effect. In contrast, the surface roughness and hardness of the specimens with surface-machined bionic fabrics were enhanced, and the wear depth of the specimens with bionic textures was significantly reduced compared to those without textures [30]. This is attributed to the fact that the surface’s bionic texture serves as a storage space for wear debris, effectively preventing texture failure, reducing frictional resistance, and significantly decreasing wear [31]. The short-tailed shrew claw texture protects the oxide layer and the soil film formed on the friction surface, inhibiting oxidation wear and fatigue wear. Secondly, due to its special surface structure, the short-tailed shrew claw texture reduces the friction heat effect of the friction surface and possesses certain animal claw friction-reduction and wear-resistance characteristics.

### 3.5. Wear Mechanism Analysis

**Figure 14:**
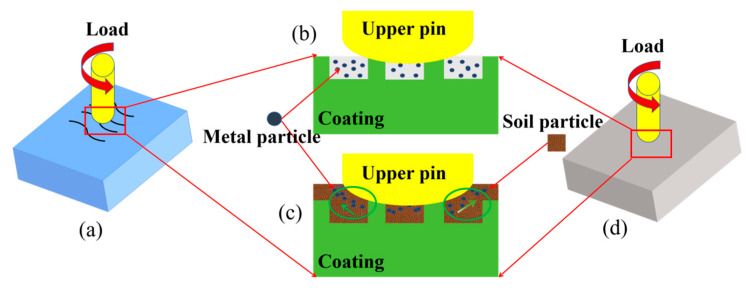
Wear mechanism diagram: (**a**) laser texture, (**b**) dry friction, (**c**) soil friction, (**d**) no texture.

The mechanistic processes by which wear occurs on 65Mn steel are demonstrated in Figure 14. In the figure, a scenario involving 65Mn steel subjected to dry friction with an unblemished surface presents a unique dynamic where direct contact is established between the upper and lower specimens. From this interaction, one may observe that the induced localized plastic deformation occurs microscopically. Such microscopic deformations result in the abrasive particles discerned at said level. External forces act upon these particles—pressure encapsulates them between the pin and the sample surface—and scratching transpires along the surface. Observably, as sliding persists, grooves appear and become increasingly perceivable. If the abrasive particles are not removed in a timely manner, they may further participate in the abrasive wear process, thereby increasing the wear on the 65Mn steel surface (Figure 14a). A bionic texture can effectively retain the abrasive particles and ensure that the contact stress of the friction vice is not too high, avoiding texture failure and significantly reducing the abrasive wear at the friction interface. Figure 14c presents the mechanism of wear that prevails in soil friction scenarios: serving as an interstitial medium, soil particulates facilitate the removal of abrasive residues, thereby mitigating the incisive influence exerted by metal particulates upon the protective layer. Observe that abrasion imprints appear on the substrate’s exterior and a portion of the diminutive soil granules move into the friction path, effectuating a filling function. This occurrence delineates a reduction in the area of contact situated between the pin and the specimen, operating in a cushioning capacity and reducing wear [32].

## 4. Conclusions

(1) Compared to specimens lacking a bionic texture, those with bionic textures of varying densities applied to their surfaces exhibit superior friction and wear performance. Bionic surface textures can accumulate significant amounts of abrasive material within their surface grooves, with only minimal amounts of abrasive debris adhering to the friction sub-surfaces. This demonstrates that texturing effectively serves to collect abrasive particles.
(2) The friction coefficient was minimized at a distribution density of 20% in dry friction conditions, and it was reduced by 24% compared to the non-bionic texture. Under soil friction conditions, the coefficient of friction was minimized at a distribution density of 30%, and it was reduced by 2.9% compared to the non-bionic texture. This indicates that the processing of a bionic texture on the surface of a specimen can significantly decrease its frictional resistance.
(3) The wear of specimens with bionic textures was significantly reduced compared to those without bionic textures under both conditions. Specifically, wear was reduced by 10.6% at a distribution density of 30% under dry friction conditions and by 19.7% at the same distribution density under soil friction conditions.
(4) Under conditions of soil friction, applying a bionic texture to the surfaces of specimens can effectively reduce frictional wear and enhance the friction wear performance of implements that come into contact with earth. This has excellent potential for application in the agricultural machinery industry in the future.
(5) The texture of the short-tailed shrew’s claw has significant advantages in terms of friction and wear resistance, especially in the field of agricultural machinery, where it has enormous application potential.

## Figures and Tables

**Table 1:** Chemical composition of 65Mn steel.

Element	Mn	C	Cr	Cu	Ni	Si	S	P
Content	0.90–1.20	0.62–0.70	≤0.25	≤0.25	≤0.25	0.17–0.37	≤0.035	≤0.035

**Table 2:** Equations for fitting the inner and outer curves of each claw toe.

Claws and Toes	Fitted Equation	Coefficient of Determination (CD)
Toe 1 inside	y_1n_ = 0.15571 + (−64.50373 * x) + 6695.13117 * x^2^	R^2^ = 0.95765
Toe 1 outside	y_1w_ = 0.01986 + (−8.61638 * x) + 935.60284 * x^2^	R^2^ = 0.99609
Toe 2 inside	Y_2n_ = 0.00461 + (−2.86875 * x) + 449.06665 * x^2^	R^2^ = 0.98304
Toe 2 outside	Y_2w_ = 0.00905 + (−5.00498 * x) + 684.30525 * x^2^	R^2^ = 0.97772
Toe 3 inside	Y_3n_ = 0.008 + (−3.8001 * x) + 504.86579 * x^2^	R^2^ = 0.99842
Toe 3 outside	Y_3w_ = 0.01999 + (−8.50617 * x) + 951.82364 * x^2^	R^2^ = 0.99643

**Table 3:** Grace X 355-3 Alaser laser parameters.

Equipment	Optical Power	Frequency	Wavelength	Pulse
Grace X 355-3 Alaser	3 W	30 kHz	355 nm	25 Ms

**Table 4:** Chemical composition of GCr15 steel.

Element	C	Cr	Mn	Si	P	S	Ni	Cu
Content	0.95–1.05	1.30–1.65	0.2–0.40	0.15–0.35	≤0.027	≤0.020	≤0.030	≤0.025

**Table 5:** Soil particle size.

Particle Size (µm)	2000	2000–1000	1000–500	500–100	<100	Total Weight
Measured value (g)	124.301	96.538	64.909	111.594	66.551	463.89
Specific gravity	26.795%	20.810%	13.992%	24.056%	14.346%	100%

**Table 6:** Operating conditions.

Conditions	Rotation Radius	Load	Rotational Speed	Duration	Texture Density			
Dry	10 mm	10 N	200 rpm	120 min	0	20%	25%	30%
Soil								

## Data Availability

The data supporting this study’s findings are available from the corresponding author upon reasonable request.
